# Case Report: Synchronous primary location of gastrointestinal stromal tumors (GIST) and adenocarcinoma of the colon: an unusual occurrence

**DOI:** 10.12688/f1000research.139536.2

**Published:** 2025-02-26

**Authors:** Asma Sghaier, Amine El Ghali, Khalil Fradi, Dorra Chiba, Fehmi Hamila, Sabri Youssef

**Affiliations:** 1 General surgery, Faculty of Medicine, University of Sousse, Sousse, 4000, Tunisia; 2 General surgery, Hospital Farhat Hached, Sousse, Tunisia; 3 Cytogenetic and anatomopathology, Hospital Farhat Hached Sousse, Sousse, Tunisia

**Keywords:** Colon- adenocarcinoma-GIST- C-Kit-synchronous

## Abstract

**Background:** We have little knowledge about the synchronous occurrence of gastrointestinal stromal tumors (GISTs) and other types of histologic tumors. This association is very rare.

**Case presentation:** We describe a case of synchronous stromal tumor and adenocarcinoma of the left side colonic localization. Immunohistochemistry identified c-Kit expression. The discovery of colonic adenocarcinoma was on operative specimen after histologic examination. The patient underwent left carcinologic colectomy with stoma. Follow-up at one year postoperatively did not detect tumor recurrence.

**Discussion:** Clinical implications of the association between these two neoplasms are not clearly described. Treatment depends on the dominance of one histologic type. Knowledge of the genetic data of this association offers opportunity of treatment with the new targeted-therapy molecules. Surgical resection, may remain the curative treatment.

**Conclusions:** Synchronous adenocarcinoma and GIST has been more commonly described in the stomach.  The pathogeneses of tumorigenesis may not be the same for the two tumors. More studies seem be necessary to clarify a potential role of different genes in the development of adenocarcinomas. And therefore, above all their therapeutic implications

## Introduction

Stromal tumors are the most common mesenchymal tumors of the gastrointestinal tract. They derive from the interstitial cells of Kajal. ^1^ The coexistence of gastrointestinal stromal tumors (GIST) and colorectal adenocarcinomas is unusual. This association has been rather more described in the stomach. Most of them were discovered during surgical intervention for primary gastrointestinal adenocarcinoma. ^1^^,^^2^ The synchronous occurrence of primary colonic adenocarcinoma and stromal tumors brings us to think about the possibility of similar origin and carcinogenetic process, and the possibility of similar systemic drugs specially target therapy. Furthermore, the association of specific tumors often leads to the discovery of novel genetic pathways to carcinogenesis that could be important for the development of oncologic therapeutics protocols.

## Case presentation

**Figure 1.**
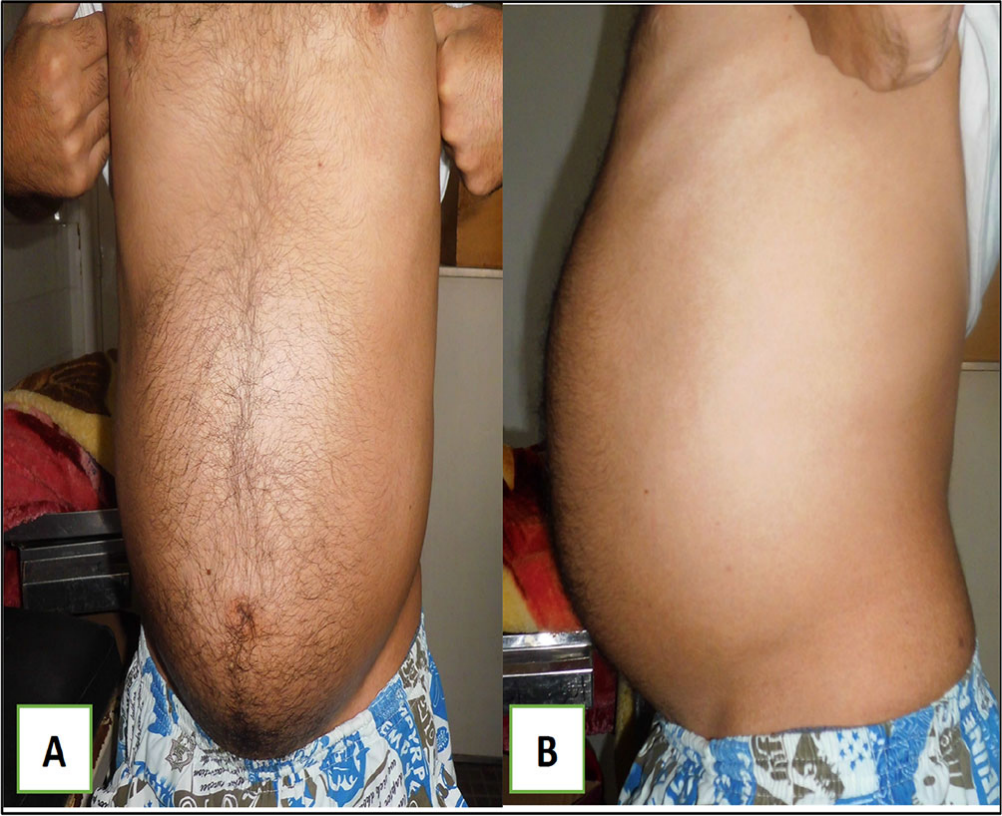
Images showing the abdominal mass in frontal and profile positions.

A 79-year-old White retired school-teacher male was admitted complaining of asthenia and diffuse abdominal pain. The patient had no notable pathological history and had never been operated on. The patient also had no familial pathologic history notably no cancer history. The physical examination revealed a large, solid pelvic mass extending to the epigastrium, which was responsible for abdominal pain and a feeling of tightness ( Figure 1).

**Figure 2.**
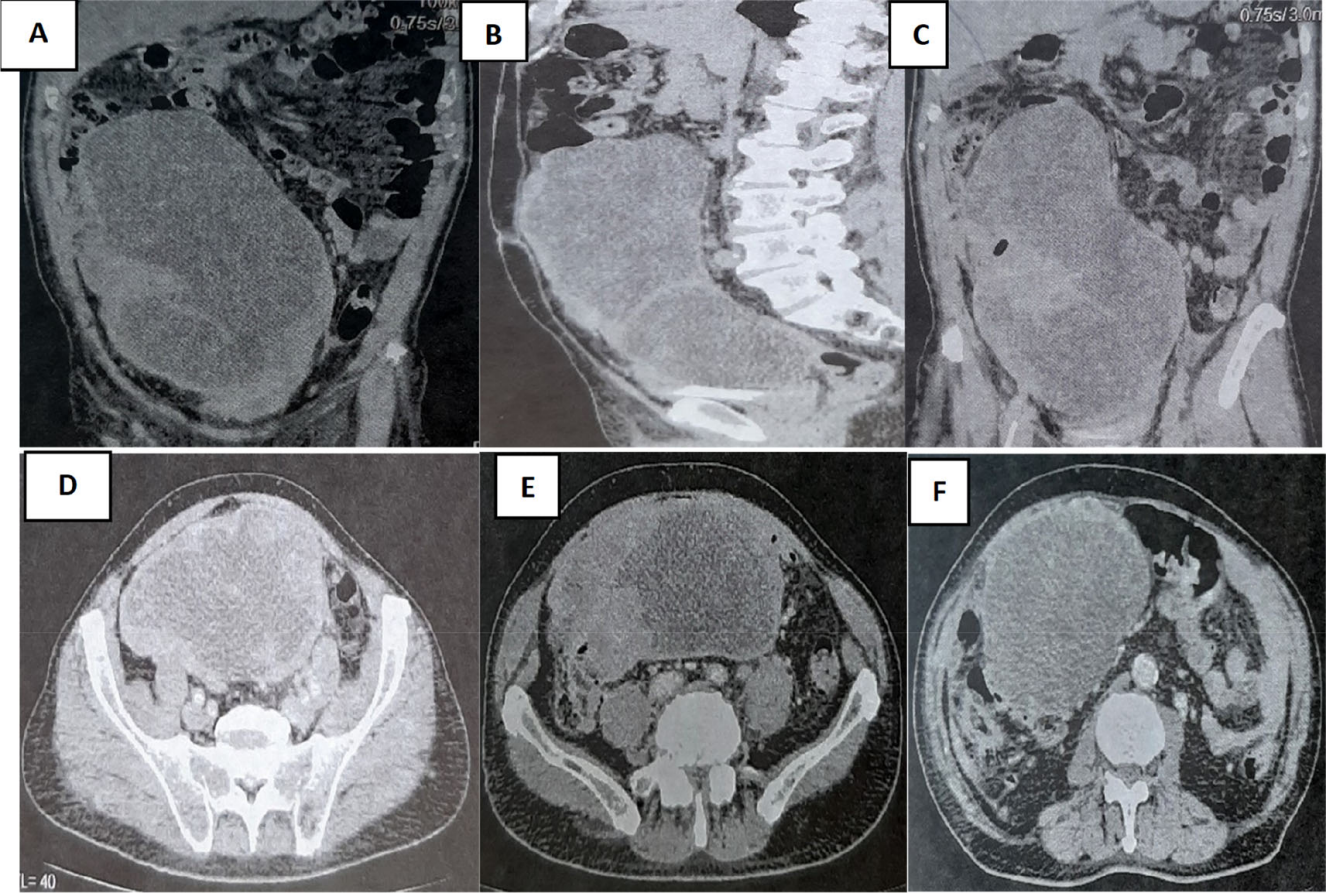
CT scan showing tumor mass.

A colonoscopy was performed but was incomplete due to the presence of an impassable stenosis at the sigmoid, which seemed to be extrinsic. An abdominal-pelvic CT scan was performed and described a large abdominal-pelvic mass of 25 cm in length, which was enhanced after injection of contrast product and seemed to have a digestive origin ( Figure 2).

**Figure 3.**
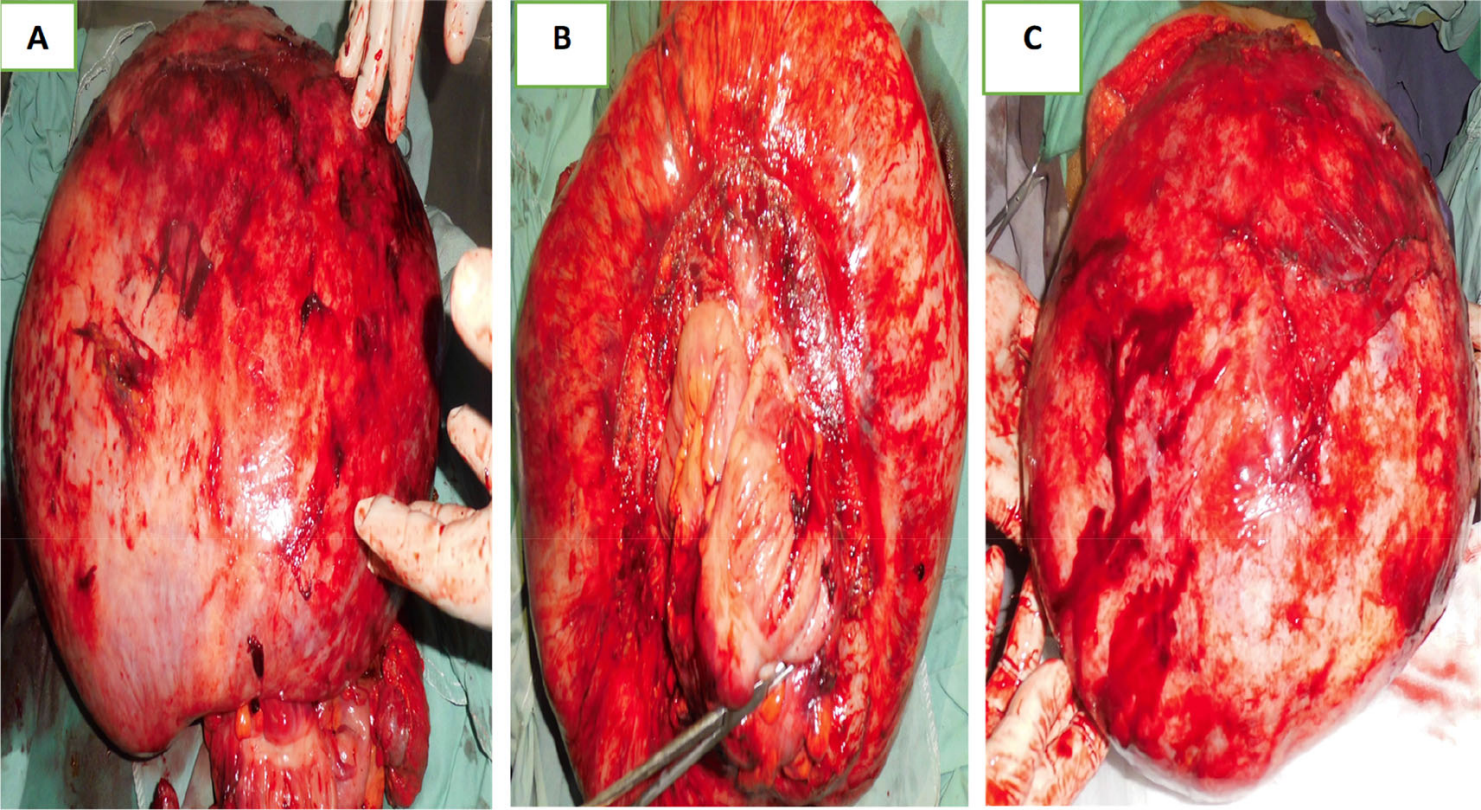
Surgical specimen.

Laparotomy confirmed the presence of a voluminous mass of the sigmoid adhering and invading the bladder dome extended to the upper rectum. This mass was friable, necrotic in places and centered by a liquefied hematoma ( Figure 3).

There was no evidence of metastatic disease. The patient had undergone extensive carcinologic resection and the bladder dome was partially resected. In fact, a bladder bezel that was adhering to the tumor was removed. Given the hemorrhagic nature and the precarious nutritional state of the patient. We decided to postpone an anastomosis and perform a Hartmann stoma. Surgical follow-up was favorable, and the patient was discharged on the eighth day of the post-operative period.

**Figure 4.**
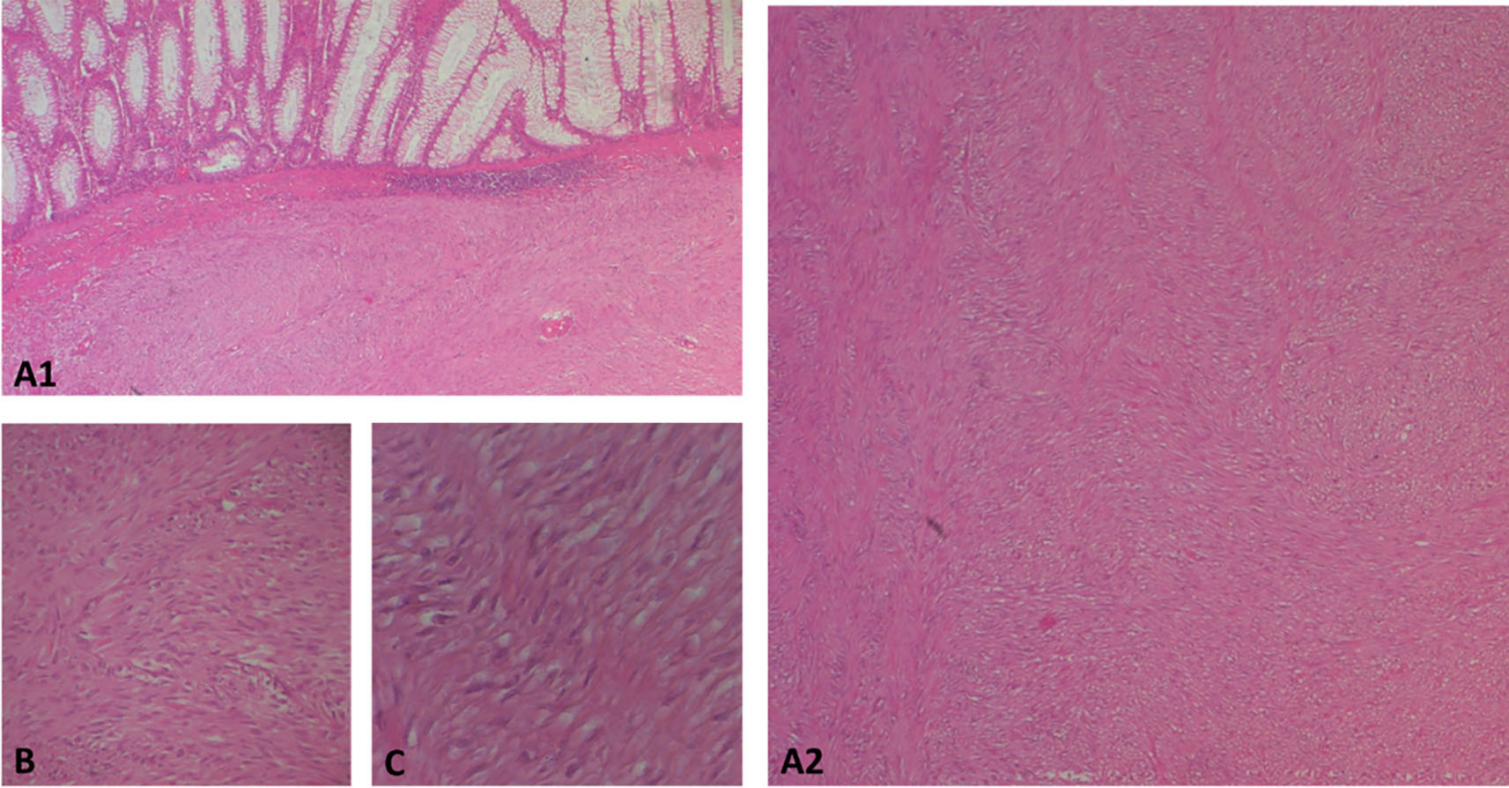
Subtype of gastrointestinal stromal tumour (GIST): A(1and2): photomicrographs showing at low magnification (HE*40) a proliferation with a disorganized bundle architecture. B and C: at higher magnification *100 and *400 respectively show spindle cells with eosinophilic cytoplasm and elongated hyperchromatic nuclei that are not very atypical.

The surgical resection piece was sent to the department of Pathology, macroscopically, the specimen corresponded to the left colon extended to the rectum measuring 20 cm in length, 3.5 cm at the colonic border and 2.5 cm at the rectal border. The wall was the site of a shredded tumor lesion extending 15 cm in height ( Figure 4).

On opening, the colonic lumen was partially obstructed by a 4 cm high protrusion of the colonic mucosa, under which there was a whitish tumor proliferation with two macroscopic aspects, whitish fasciculated in the submucosa ulcerating the mucosa ( Figure 4).

This aspect is partially separated by the muscularis propria from the other aspect of the tumor, which shows necrotic and hemorrhagic remodeling, and extends towards the serosa, where there is a capsular rupture.

At 1.5 cm from this tumor there was an intraluminal polypoid lesion measuring 1.5 cm long.

**Figure 5.**
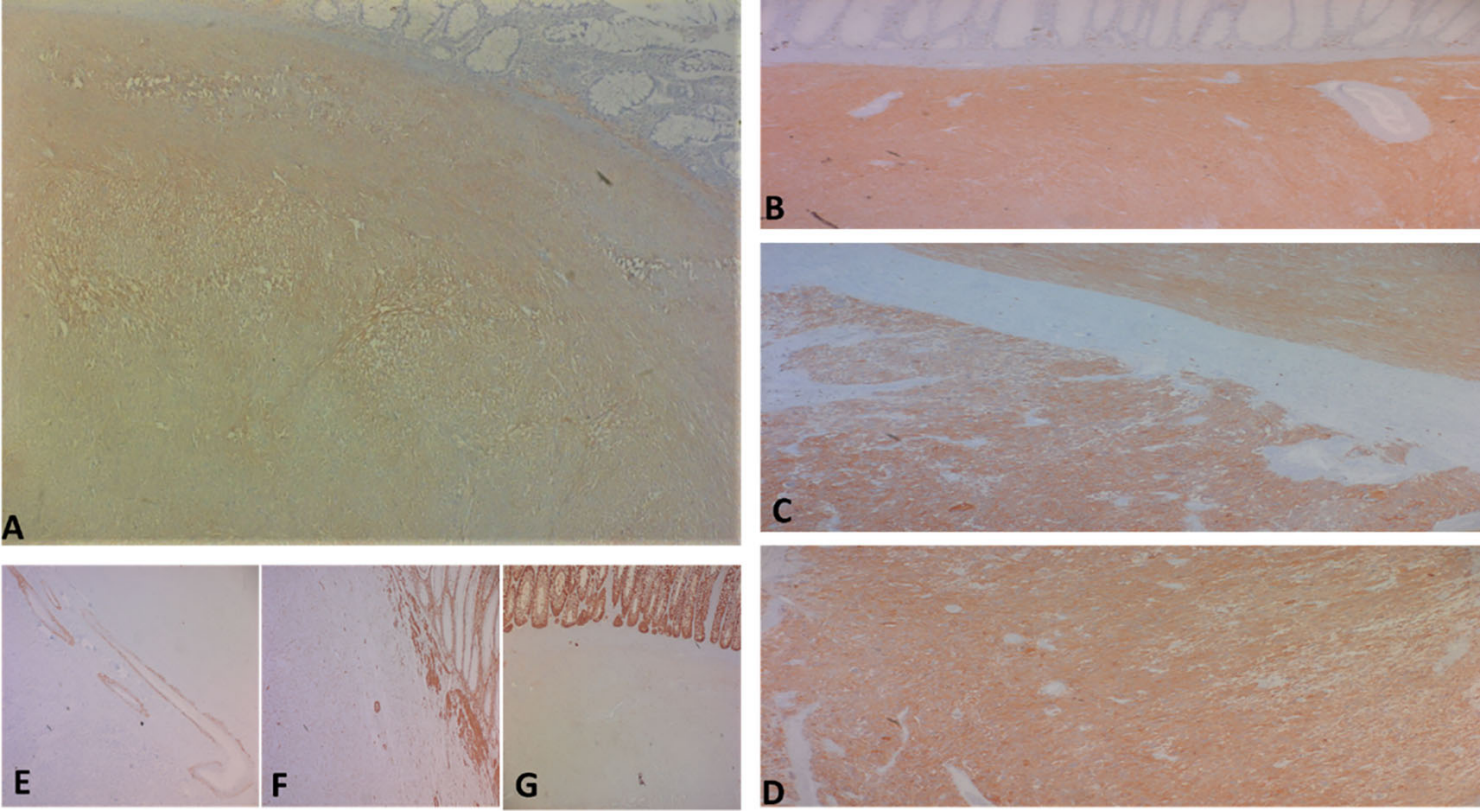
The tumor cells are positive for C-kit (A) and Dog-1 (B: fusiform contingent, C: interface of the two contingents, D: epithelial contingent). They are negative for Desmin (E), AML smooth muscle actin (F) and panCytokeratin (G).

Regarding histology, the main tumor was a mixed gastrointestinal stromal tumor (GIST), with spindle cells in the submucosa and epithelial cells in the outer layers of the colonic wall, with a high risk of recurrence due to the innumerable mitoses, which exceeded 100 mitoses per 50 fields at high magnification, and the capsular rupture, according to the Miettinen and Joensuu classificationv ( Figure 5) and was classified pT4 according to TNM 2017 in the eighth edition. ^3^

**Figure 6.**
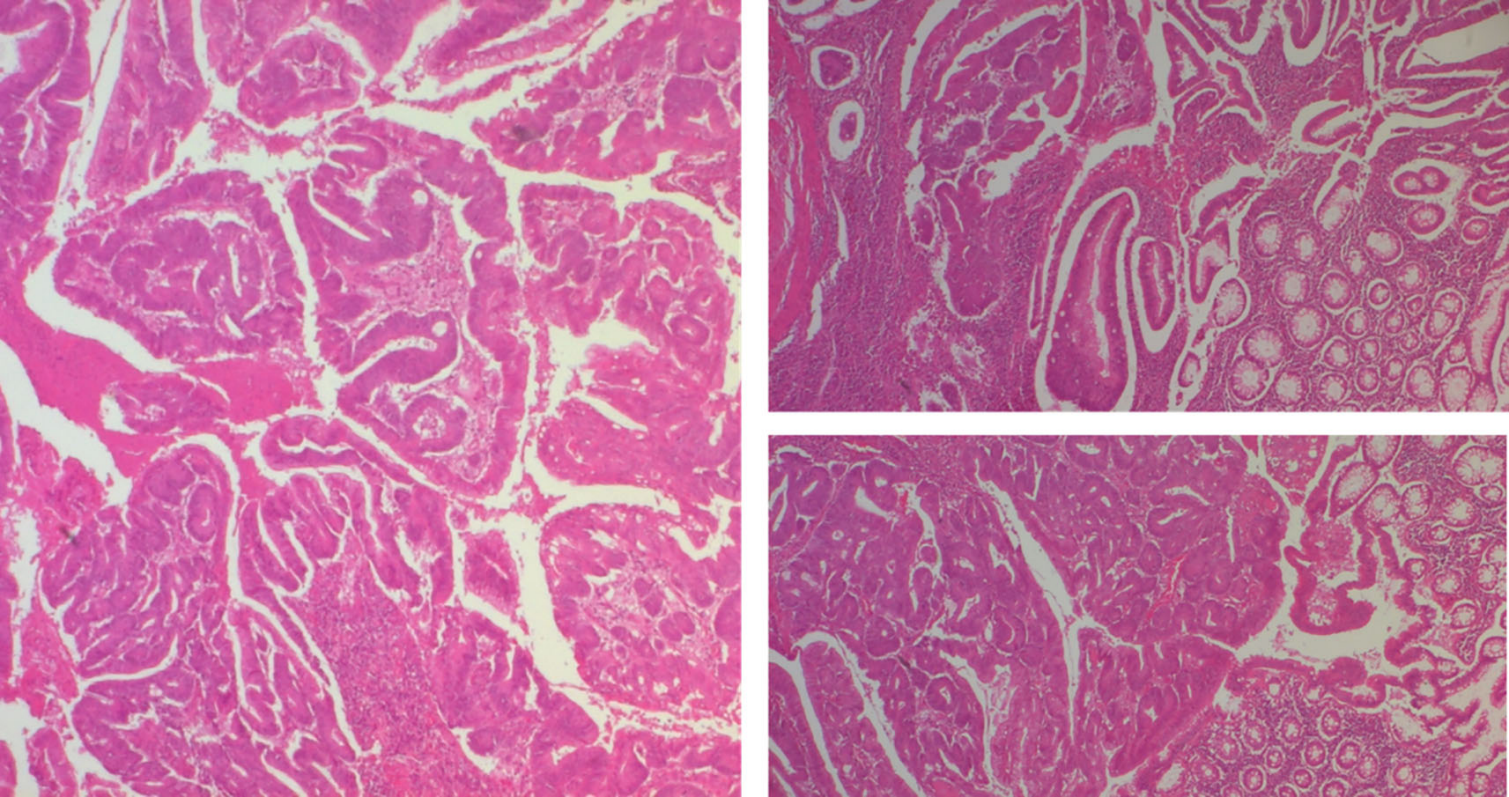
A well-differentiated, low-grade NOS-type adenocarcinoma invading the submucosa without going beyond it.

The polypoid lesion was an adenocarcinoma NOS type well differentiated developed on degenerated adenoma stadified pT1N0 ( Figure 6).

The patient medical file was discussed with the multidisciplinary consensus staff indicated treatment with imatinib-based targeted therapies. Follow-up at one year postoperatively did not detect tumor recurrence.

## Discussion

Stromal tumors are the most common mesenchymal tumors of the digestive tract. Yearly incidence rates range between 4.3 to 22 per million in the world, which is due to variability, the improving diagnostic criteria and a lack of GIST registries. ^4^ Simultaneous presence of Colonic adenocarcinoma and stromal tumor is an uncommon occurrence. Because of the high incidence of adenocarcinoma histological type and the frequency of gastrointestinal stromal tumors (GIST), a fortuitous relationship based on the available data cannot be ruled out. The Genetic pathways of tumorigenesis are different for the two histologic types; c-Kit appears to be occasionally expressed in adenocarcinoma, and there is no evidence if the protein is indeed in the carcinogenetic process; this report is not available for stromal tumors. A review concluded that STI571 blocks the growth of colonic carcinoma cell lines. ^5^ These results justified by preclinical investigations of c-Kit expression in colonic cancers had as objective to evaluate the use of tyrosine kinase inhibitors in the treatment of colorectal carcinomas.

We have presented a case of synchronous invasive colonic GIST with adenocarcinoma. Despite the relative common occurrence of GISTs, reports of synchronous adenocarcinoma and GISTs are quite rare. According to Kover *et al*., 7 of 43 patients with histologically evidence of GISTs were found with second histological type; three of these GISTs were colorectal adenocarcinomas. ^5^ A second study realized by Au *et al*., found that nearly 41% of the stromal tumors were synchronous association with second malignant tumor, and 38% of these second malignancies were intestinal. ^6^

Colonic adenocarcinoma and GIST present evidence of familial predisposition, except hereditary cancers. In another case, the patient did not have a family history of gastrointestinal or other malignancies. ^7^ The genetic polymorphism of these two histologic types has been particularly investigated. Through progression from normal colonic epithelium to adenoma and adenocarcinoma, various genetics cancers can occur. ^8^

Mechanisms have been clarified in sporadic colorectal cancer: chromosomal instability is responsible for 85% of the whole cases, and microsatellite instability, in the rest 15%. ^8^ Unusually, none of the most commonly involved genes in colorectal carcinogenesis (APC, DCC, p53, K-ras, DNA mismatch repair genes) have been identified to be associated in the pathway of stromal tumors. Nevertheless, the GISTs seem to be related with the proto-oncogene mutation c-Kit, a tyrosine kinase receptor during embryonic growth and on postnatal. Activation of c-Kit by its ligand, SCF, may generates a cascade of cellular process involving transformation, differentiation, cell proliferation, adhesion, and chemotaxis. ^9^

When it is possible, surgery is the ideal therapeutic alternative with curative intention for non-metastatic stromal and adenocarcinoma at the same time. The operative strategies are in most situations wide and extensive. ^10^ Since nodal involvement is rare, lymph-node clearance is not recommended. ^11^ The prognosis of stromal tumors depends on tumor localization, its size, and the mitotic activity. ^12^^,^^13^ The stage of synchronous malignancies is crucial because the dominant one is responsible of the outcome and survival. ^13^ Imatinib provide special focus in the treatment of stromal tumors; particularly, for neoadjuvant process. The benefits of this target therapy are well established to downstage inoperable cases especially by decreasing size. As a result, safe resection margins and therefore an R0 resection are recommended. ^14^

GIST presents a high rate of recurrence (40% within 2 years). ^15^ Just such colonic carcinoma, GISTs usually metastasize to the liver. ^16^ Overall survival after complete resection of stromal tumors ranges from 47% to 66% at 5 years, and seems to be longer in patients with low-grade tumors: 100% at 10 years for tumors with 0–1 mitosis/30 hpf. High-grade lesions:>10 mitosis/10 hpf, have the worst outcome: 0% survival at 10 years. Nevertheless, the absence of a high mitotic index does not guarantee a better outcome. ^17^ Overall, a 5-year survival for colonic adenocarcinoma correlates with the preoperative staging, and ranges from 3–8% for stage IV to 90% for stage I. ^18^

For our case the predominant histologic type was the stromal one (GIST), with a high risk of recurrence due to the innumerable mitoses, which exceeded 100 mitoses per 50 fields at high magnification, and the capsular rupture, according to the Miettinen and Joensuu classification and was classified pT4 according to TNM 2017.

The adenocarcinoma type was well differentiated developed on degenerated adenoma stadified pT1N0. The patient was treated with imatinib-based targeted therapies.

Eticulous immunohistochemical and molecular biology study of all resection specimens are highly recommended whenever the combination of two histological types is found in the primary anatomopathological study.

These in-depth and ideally exhaustive studies guarantee the development of new targeted therapies and immunotherapies that would provide these patients with the opportunity of complete remission.

Nonetheless, all such cases must be discussed at a multidisciplinary concertation involving all the medical staff.

Finally, this case certainly illustrates a rare association of two histological entities. There are few cases described in the literature, which limits the possibility of reaching well-codified conclusions regarding management.

However, we believe that this case highlights the necessity for more thorough immunohistochemical and molecular biology studies.

The aim is to draw up recommendations with a high level of scientific evidence.

## Conclusion

Synchronous tumors rare cancer of the colonic with the co-existence of two histologically different neoplasms occurring in the same site without contact between them. This condition is rarely proven in preoperative investigations. Lymph node dissection is essential and must be performed according to the relevant guidelines for oncology, in the absence of histological evidence as to the most significant histological type. Perfect histopathologic examination with multiple biopsies and pathologic examination of resection specimens is required to detect synchronous tumors. Those with advanced or aggressive behavior has pejorative prognostic significance and should receive adjuvant therapy.

Pathogeneses of such association are still not yet well identified. More studies are required to understand this incident to provide optimal curative management for patients. Sophisticated molecular biology studies are the bridge to innovative, more effective and targeted therapies.

## Consent

Written informed consent was obtained from the patient for publication of this case report and accompanying images.

## Data Availability

All data underlying the results are available as part of the article and no additional source data are required.
