# Supplementary material for: Case Report: Synchronous primary location of gastrointestinal stromal tumors (GIST) and adenocarcinoma of the colon: an unusual occurrence
Source: F1000Res. 2025 Oct 6;12:1055. Originally published 2023 Aug 30. [Version 4] doi: 10.12688/f1000research.139536.4 (PMC12538204; doi:10.12688/f1000research.139536.4)
Supplement: Peer review history

# Reviewer report: Reviewer response for version 4

**DOI:** 10.5256/f1000research.188933.r420585

**Published:** 2025-10-20

**Authors:** Nafiza Martini

**Affiliations:** 1 University of Illinois Chicago College of Medicine, Chicago, Illinois, USA

Thank you very much for your thoughtful and detailed revisions. I appreciate how comprehensively you addressed each point, particularly the enhanced molecular discussion and the addition of evidence-based management recommendations. The revised version reflects clear improvement in scientific depth and clinical applicability.

 Kind regards,

 Dr. Nafiza Martini

Are enough details provided of any physical examination and diagnostic tests, treatment given and outcomes?

Yes

Is the case presented with sufficient detail to be useful for other practitioners?

Yes

Is sufficient discussion included of the importance of the findings and their relevance to future understanding of disease processes, diagnosis or treatment?

Yes

Is the background of the case’s history and progression described in sufficient detail?

Yes

Reviewer Expertise:

General medicine and molecular biology of cancers

I confirm that I have read this submission and believe that I have an appropriate level of expertise to confirm that it is of an acceptable scientific standard.

# Reviewer report: Reviewer response for version 3

**DOI:** 10.5256/f1000research.179521.r401921

**Published:** 2025-09-27

**Authors:** Nafiza Martini

**Affiliations:** 1 University of Illinois Chicago College of Medicine, Chicago, Illinois, USA

This is a well-documented and instructive case on synchronous GIST and colonic adenocarcinoma, highlighting the complexity of dual malignancies and the role of multidisciplinary surgical strategies. The histopathological detail and therapeutic implications are clearly presented.

 This is an instructive and rare case that not only documents a clinical challenge but also raises important questions about shared and divergent oncogenic pathways. Highlighting these molecular aspects, along with similar reports in the literature, would strengthen the translational value of the work.

 In the Discussion section, authors have mentioned " The aim is to draw up recommendations with a high level of scientific evidence"; actually, it is the author's duty to give the recommendations after finishing the discussion. so, they should read more deeply in literature and empower the paper in a way that fills the gaps and goes deep inside the molecular mechanism with respect of focusing on the type of this paper as a clinical case report in the end!

Are enough details provided of any physical examination and diagnostic tests, treatment given and outcomes?

Yes

Is the case presented with sufficient detail to be useful for other practitioners?

Yes

Is sufficient discussion included of the importance of the findings and their relevance to future understanding of disease processes, diagnosis or treatment?

Yes

Is the background of the case’s history and progression described in sufficient detail?

Yes

Reviewer Expertise:

General medicine and molecular biology of cancers

I confirm that I have read this submission and believe that I have an appropriate level of expertise to confirm that it is of an acceptable scientific standard, however I have significant reservations, as outlined above.

# Response

**Published:** 2025-09-27

**Authors:** Asma SGHAIER

**Affiliations:** GENERAL SURGERY, FACULTY OF MEDICINE OF SOUSSE, SOUSSE, Tunisia

Dear Dr. Martini,

 We are writing to thank you sincerely for your careful and insightful review of our manuscript, "Synchronous primary location of gastrointestinal stromal tumors (GIST) and adenocarcinoma of the colon: an unusual occurrence." We greatly appreciate your affirmation of the case's instructional value and its clear histopathological detail.

 We concur completely with your reservations regarding the need to strengthen the translational value, deepen the molecular discussion concerning shared oncogenic pathways, and, most importantly, provide explicit, evidence-based management recommendations. Your comments have been instrumental in significantly enhancing the scientific rigor and clinical utility of our case report.

 The revised manuscript (Version 3) has been meticulously updated, with all changes highlighted in **bold** text, to incorporate your expert recommendations.

 Below is our point-by-point response detailing the revisions made:

 Reviewer Comment

***Authors’ Response* "Highlighting these molecular aspects, along with similar reports in the literature, would strengthen the translational value of the work."** (Referring to shared and divergent oncogenic pathways).We fully agree that a deeper molecular discussion is crucial for the translational value of this rare co-occurrence. We have significantly revised the **Discussion** section to address the potential molecular link between GIST and colorectal adenocarcinoma (CRC). Specifically, we now elaborate on the **convergence of oncogenic signaling at the RAS/MAPK (Mitogen-Activated Protein Kinase) pathway** .

 We argue that while the primary drivers are distinct (KIT/PDGFRA mutations in GIST vs. APC/KRAS mutations in CRC), a shared functional reliance on the hyperactivation of this ubiquitous proliferative cascade may facilitate co-tumorigenesis. This concept moves the analysis beyond a purely incidental co-occurrence. We further strengthen this argument by discussing **Neurofibromatosis Type 1 (NF1)** as a clear biological paradigm where NF1 inactivation leads to uncontrolled RAS signaling, thereby predisposing patients to both GIST and CRC .

**"In the Discussion section, authors have mentioned 'The aim is to draw up recommendations with a high level of scientific evidence'; actually, it is the author's duty to give the recommendations after finishing the discussion."**We appreciate your guidance on the responsibility of the authors to provide definitive conclusions.

 We have removed the aspirational sentence and, instead, have added a new, clearly delineated subsection titled  (Evidence-Based Clinical Management Recommendations).

 This section explicitly details the necessary surgical and adjuvant protocols, guided by the most aggressive entity present—the **Very High-Risk GIST** component—thereby fulfilling our duty to provide clear, evidence-based clinical guidance.  

**"...empower the paper in a way that fills the gaps and goes deep inside the molecular mechanism with respect of focusing on the type of this paper as a clinical case report in the end!"**To empower the manuscript and deepen the discussion on molecular mechanisms, we have integrated an emphasis on the **imperative of comprehensive molecular profiling** for both lesions. This includes sequencing the GIST for KIT/PDGFRA and the CRC for KRAS/BRAF status, a critical step for understanding the full activation status of the MAPK pathway and for future therapeutic considerations.

 The final **Conclusion** has also been refined to underscore that the **shared functional reliance on the RAS/MAPK signaling cascade provides a compelling molecular mechanism** that warrants further targeted investigation.  

 We believe these extensive revisions significantly enhance the scientific depth of the manuscript, particularly concerning the molecular oncology and the practical clinical implications of this rare synchronous malignancy.

 Thank you once again for your invaluable input. We hope that the revised manuscript now meets the high standards of scientific evidence and clarity expected for publication.

 Sincerely,

 Authors Dr Asma SGHAIER

 On behalf of all authors,

# Reviewer report: Reviewer response for version 3

**DOI:** 10.5256/f1000research.179521.r376373

**Published:** 2025-04-22

**Authors:** Shamus R. Carr

**Affiliations:** 1 National Cancer Institute, National Institutes of Health, Bethesda, Maryland, USA

I have read and reviewed version 3 of this manuscript. I wish to thank the authors for their careful review of the prior comments and their attention to addressing them with thoughtful edits and clarifications. This case report is now acceptable for indexed.

Are enough details provided of any physical examination and diagnostic tests, treatment given and outcomes?

Partly

Is the case presented with sufficient detail to be useful for other practitioners?

Partly

Is sufficient discussion included of the importance of the findings and their relevance to future understanding of disease processes, diagnosis or treatment?

Partly

Is the background of the case’s history and progression described in sufficient detail?

Yes

Reviewer Expertise:

NA

I confirm that I have read this submission and believe that I have an appropriate level of expertise to confirm that it is of an acceptable scientific standard.

# Reviewer report: Reviewer response for version 2

**DOI:** 10.5256/f1000research.178351.r368590

**Published:** 2025-03-14

**Authors:** Shamus R. Carr

**Affiliations:** 1 National Cancer Institute, National Institutes of Health, Bethesda, Maryland, USA

This case report highlights a unique finding of a patient with synchronous GIST tumor of the colon and colonic adenocarcinoma. Supposedly this is a revision, but none of the simple editorial recommendations that were previously made have been addressed from the peer review I submitted on October 18, 2023 (Points 1-4 and Points 7-12)5.  Therefore, I am resubmitting the points below that need to be addressed before publication. It is also possible that the revision you updated somehow is not what I am able to see.  Please either address the points below or provide me with the revision where all the points below are addressed. Two points, one about the PET scan and the other about if the tumor was tested for SDHA deficiency were adequately addressed.

 Points:

1. In the Abstract, the work “carcinologic” is used. The root of this work “carcinology” means “study of crustaceans”. This word is therefore incorrect and either should be deleted or changed.
2. Please add a “period” at the end of the last sentence in the Conclusions of the Abstract.
3. The term “dominance of one histologic type” is unclear as it is known that GIST arise from the stroma and adenocarcinoma of the colon arise from the mucosal lining. This also has implications on distant spread. Could the authors rewrite this sentence? I think they mean “the more aggressive or higher stage”, but I may be wrong.
4. GIST tumors are thought to arise from the interstitial cells of Cajal (not Kajal). Named after the Spanish neuropathologist and Nobel laureate Ramón y Cajal.
5. Thank you for the answer about the PET scan. I understand the point about getting this type of study in some countries.
6. I figure SHEA is a typo for “SDHA deficient GIST tumor”.
7. “Colonic” does not need to be capitalized unless it is the first word of a sentence.
8. The last sentence of the third paragraph of in the Conclusions section is unclear. Can this be rewritten for clarity?
9. In the 5 ^th^ paragraph of the Conclusions section, the authors imply that lymphadenectomy is not required for colon cancer. This is clearly not what the authors intend. Please review and rewrite to clarify.
10. In the 6 ^th^ paragraph of the Conclusions section, the second sentence needs a small edit. It should read “Just like…” and not as written, “Just such…”.
11. When mentioning the TNM staging system update from 2017 for GIST tumors, it is probably better to state the edition of the AJCC staging (which I believe was the 7 ^th^ AJCC edition, but I would double check).
12. Would the authors consider rewriting the last 7 paragraph of the Conclusions section into one or two paragraphs?

Are enough details provided of any physical examination and diagnostic tests, treatment given and outcomes?

Partly

Is the case presented with sufficient detail to be useful for other practitioners?

Partly

Is sufficient discussion included of the importance of the findings and their relevance to future understanding of disease processes, diagnosis or treatment?

Partly

Is the background of the case’s history and progression described in sufficient detail?

Yes

Reviewer Expertise:

NA

I confirm that I have read this submission and believe that I have an appropriate level of expertise to confirm that it is of an acceptable scientific standard, however I have significant reservations, as outlined above.

# Response

**Published:** 2025-03-16

**Authors:** Asma SGHAIER

**Affiliations:** GENERAL SURGERY, FACULTY OF MEDICINE OF SOUSSE, SOUSSE, Tunisia

Dear Shamus R. Carr

 Thank you for your letter and for the ’ comments concerning our manuscript.

 I would like to express my gratitude once again for the great care you have taken and for the time allocated to review our manuscript.

 Thank you very much for your consideration, and we really appreciate the comments and have learned a lot. Appropriate changes were made according to the suggestions of reviewers and editor.

 Those comments are all valuable and very helpful for revising and improving our paper, as well as the important guiding significance to our researches. We have studied comments carefully and have made correction which we hope meet with approval. Revised portion are marked in red in the paper. The main corrections in the paper and the responds to the Editor’s and reviewer’s comments are as flowing.

 1-In the Abstract, the work “carcinologic” is used. The root of this work “carcinology” means “study of crustaceans”. This word is therefore incorrect and either should be deleted or changed.

 Response: the term mentioned has been removed and substituted with the correct jargon

 2- Please add a “period” at the end of the last sentence in the Conclusions of the Abstract.

 Response: We have adjusted as requested.

 3- The term “dominance of one histologic type” is unclear as it is known that GIST arise from the stroma and adenocarcinoma of the colon arise from the mucosal lining. This also has implications on distant spread. Could the authors rewrite this sentence? I think they mean “the more aggressive or higher stage”, but I may be wrong.

 Response: the appropriate description has been provided in the manuscript in accordance with your well-founded proposal.

 4- GIST tumors are thought to arise from the interstitial cells of Cajal (not Kajal). Named after the Spanish neuropathologist and Nobel lureate Ramón y Cajal.

 Response: We have indeed revised the name.

 5- I figure SHEA is a typo for “SDHA deficient GIST tumor”.

 Response: your comment is well-founded.

 6-“Colonic” does not need to be capitalized unless it is the first word of a sentence.

 Response: Your comment is well-founded, and we have made the necessary corrections. I congratulate you on your careful analysis.

 7- The last sentence of the third paragraph of in the Conclusions section is unclear. Can this be rewritten for clarity?

 Response: we have removed the expression that gives rise to the lapse of understanding and ambiguity.

 8- In the 5th paragraph of the Conclusions section, the authors imply that lymphadenectomy is not required for colon cancer. This is clearly not what the authors intend. Please review and rewrite to clarify.

 Response: Lymph node dissection is essential and must be performed according to the relevant guidelines for oncology.

 10-In the 6th paragraph of the Conclusions section, the second sentence needs a small edit. It should read “Just like…” and not as written, “Just such…”.

 Response: We have inserted the recommended term.

 11- When mentioning the TNM staging system update from 2017 for GIST tumors, it is probably better to state the edition of the AJCC staging (which I believe was the 7th AJCC edition, but I would double check).

 Response: we have checked that the 2017 edition is indeed the 8th edition. However, we are awaiting the results of your research in the event of any discrepancies.

 12- Would the authors consider rewriting the last 7 paragraph of the Conclusions section into one or two paragraphs?

 Response: We have split the paragraph into two concise parts.

 We express once again our sincere appreciation and deep gratitude to all the reviewers and to the editorial board for providing us with the opportunity to revise our manuscript and to improve the quality of our submission.

# Reviewer report: Reviewer response for version 1

**DOI:** 10.5256/f1000research.152818.r215037

**Published:** 2023-10-20

**Authors:** Shamus R. Carr

**Affiliations:** 1 National Cancer Institute, National Institutes of Health, Bethesda, Maryland, USA

This case report highlights a unique finding of a patient with synchronous GIST tumor of the colon and colonic adenocarcinoma. There are areas where some editing would enhance the clarity of the text. The authors should consider offering some thought or recommendations on how they might further delve into the question of common carcinogenic pathways.

 Points:

1. In the Abstract, the work “carcinologic” is used. The root of this work “carcinology” means “study of crustaceans”. This word is therefore incorrect and either should be deleted or changed.
2. Please add a “period” at the end of the last sentence in the Conclusions of the Abstract.
3. The term “dominance of one histologic type” is unclear as it is known that GIST arise from the stroma and adenocarcinoma of the colon arise from the mucosal lining. This also has implications on distant spread. Could the authors rewrite this sentence? I think they mean “the more aggressive or higher stage”, but I may be wrong.
4. GIST tumors are thought to arise from the interstitial cells of Cajal (not Kajal). Named after the Spanish neuropathologist and Nobel laureate Ramón y Cajal.
5. Was a PET scan done prior to surgery to evaluate for distant metastatic disease? If the case was deemed emergent, and therefore a PET would not have been able to be performed, please note that about the operation.
6. Was the GIST tumor checked to see if it was SDHA deficient?
7. “Colonic” does not need to be capitalized unless it is the first word of a sentence.
8. The last sentence of the third paragraph of in the Conclusions section is unclear. Can this be rewritten for clarity?
9. In the 5 ^th^ paragraph of the Conclusions section, the authors imply that lymphadenectomy is not required for colon cancer. This is clearly not what the authors intend. Please review and rewrite to clarify.
10. In the 6 ^th^ paragraph of the Conclusions section, the second sentence needs a small edit. It should read “Just like…” and not as written, “Just such…”.
11. When mentioning the TNM staging system update from 2017 for GIST tumors, it is probably better to state the edition of the AJCC staging (which I believe was the 7 ^th^ AJCC edition, but I would double check).

Would the authors consider rewriting the last 7 paragraph of the Conclusions section into one or two paragraphs?

Are enough details provided of any physical examination and diagnostic tests, treatment given and outcomes?

Partly

Is the case presented with sufficient detail to be useful for other practitioners?

Partly

Is sufficient discussion included of the importance of the findings and their relevance to future understanding of disease processes, diagnosis or treatment?

Partly

Is the background of the case’s history and progression described in sufficient detail?

Yes

Reviewer Expertise:

NA

I confirm that I have read this submission and believe that I have an appropriate level of expertise to confirm that it is of an acceptable scientific standard, however I have significant reservations, as outlined above.

# Response

**Published:** 2025-02-16

**Authors:** Asma SGHAIER

**Affiliations:** GENERAL SURGERY, FACULTY OF MEDICINE OF SOUSSE, SOUSSE, Tunisia

**Detailed Response to Reviewer**

**Submission date: 16/02/2025**

**Dear Professor Shamus R. Carr,**

 Thank you for your letter and for the reviewers’ comments concerning our manuscript.

 Thank you very much for your consideration, and we really appreciate the comments and have learned a lot. Appropriate changes were made according to the suggestions of reviewers and editor.

 Those comments are all valuable and very helpful for revising and improving our paper, as well as the important guiding significance to our researches. We have studied comments carefully and have made correction which we hope meet with approval.

 Modifications were made .

 The PET scan was not performed preoperatively. This is an expensive test, the reliability of which is not always available in our country.

 A thoracic-abdominal-pelvic CT scan was used to assess the extent of the disease, which did not reveal any secondary locations.

 The immunohistochemical study of a possible SHEA deficiency could not be performed because of the lack of suitable reactive chemicals in our hospital.

 We have verified the AJCC 2017 version and found that it is the eighth edition.

 The precisions required by the reviewer was conducted in concertation with all the authors.

**We express once again our sincere appreciation and deep gratitude to all the reviewers and to the editorial board for providing us with the opportunity to revise our manuscript and to improve the quality of our submission.**

**We hope, nevertheless, to receive positive feedback and acceptance of our work by allowing it to be published in this prestigious journal.**

**Sincerely**

**The corresponding author**
